# Genetic Diversity of Korean Wild Soybean Core Collections and Genome-Wide Association Study for Days to Flowering

**DOI:** 10.3390/plants12061305

**Published:** 2023-03-14

**Authors:** Hyun Jo, Bo-Keun Ha, Soo-Kwon Park, Soon-Chun Jeong, Jeong-Dong Lee, Jung-Kyung Moon

**Affiliations:** 1 Department of Applied Biosciences, Kyungpook National University, Daegu 41566, Republic of Korea; 2 Upland-Field Machinery Research Center, Kyungpook National University, Daegu 41566, Republic of Korea; 3 Department of Applied Plant Science, Chonnam National University, Gwangju 61186, Republic of Korea; 4 National Institute of Crop Science, Rural Development Administration, Wanju 55365, Republic of Korea; 5 Bio-Evaluation Center, Korea Research Institute of Bioscience and Biotechnology, Cheongju 28116, Republic of Korea; 6 Department of Integrative Biology, Kyungpook National University, Daegu 41566, Republic of Korea; 7 Agricultural Genome Center, National Academy of Agricultural Sciences, Rural Development Administration, Jeonju 55365, Republic of Korea

**Keywords:** core collection, flowering days, genetic diversity, maturity, wild soybean

## Abstract

The utilization of wild soybean germplasms in breeding programs increases genetic diversity, and they contain the rare alleles of traits of interest. Understanding the genetic diversity of wild germplasms is essential for determining effective strategies that can improve the economic traits of soybeans. Undesirable traits make it challenging to cultivate wild soybeans. This study aimed to construct a core subset of 1467 wild soybean accessions of the total population and analyze their genetic diversity to understand their genetic variations. Genome-wild association studies were conducted to detect the genetic loci underlying the time to flowering for a core subset collection, and they revealed the allelic variation in *E* genes for predicting maturity using the available resequencing data of wild soybean. Based on principal component and cluster analyses, 408 wild soybean accessions in the core collection covered the total population and were explained by 3 clusters representing the collection regions, namely, Korea, China, and Japan. Most of the wild soybean collections in this study had the *E1e2E3* genotype according to association mapping and a resequencing analysis. Korean wild soybean core collections can provide helpful genetic resources to identify new flowering and maturity genes near the E gene loci and genetic materials for developing new cultivars, facilitating the introgression of genes of interest from wild soybean.

## 1. Introduction

Soybean (*Glycine max* [L.] Merr.) is one of the most economically important crops worldwide, whose seeds consist of 40% protein, 20% oil, and 15% soluble carbohydrates. Soybean in Western countries is mainly used to produce vegetable oils for humans and high-protein meals for livestock, whereas it has traditionally been used as foods, such as soymilk, tofu, soy sprout, fermented soy foods, and soy sauce, in Asian countries [1,2]. Cultivated soybean was domesticated ~5000 years ago from its wild progenitor (*Glycine soja* Sieb. and Zucc.) [3,4]. The genetic diversity of cultivated soybeans revealed a narrow genetic base due to the human selection of elite lines. Using wild soybean germplasms in breeding programs increases genetic diversity because of the preservation of rare alleles of traits of interest. Understanding the genetic diversity of wild germplasms is essential for determining effective strategies to improve the economic traits of soybeans. Although wild soybeans have favorable genes, growing large populations of this plant is challenging due to undesirable traits, such as lodging, viny growth habits, hard seed coats, and pod shattering [5,6].

Constructing a core subset is one of the strategies to overcome the handling of a large population of wild soybean accessions. A core subset can represent an entire population by maximizing its genetic and phenotypic variations [7]. The advantage of a core subset collection is an increased efficiency in overcoming population size, cost, and labor drawbacks compared with the evaluation of the total population. Therefore, core subsets from various crop species have been constructed based on morphological characteristics, phenotypic assessments, and genotypic information [8,9,10,11,12,13,14,15,16]. However, phenotypic observations are mainly affected by environmental factors. The utilization of molecular markers to construct a core subset directly reflects the genetic diversity of the total population [17,18]. As soybean is a short-day and photoperiod-sensitive crop, it is crucial to grow it in suitable target regions due to the narrow growth range of latitudes to achieve maximum production. Thus, soybean flowering is determined by specific night and day lengths in the transition from vegetative to reproductive growth. Soybeans are grown under different maturity group designations to match the day and night lengths at different latitudes. The optimal region for growing soybeans is one of the critical considerations for yield potential. Farmers need to consider the maturity of soybeans to determine seed production and quality at specific latitudes. There are 13 maturity groups (MGs) in the US, ranging from MG 000 to MG X, based on their latitudinal adaptation [19,20].

Broad adaptation for soybean growth is due to quantitative trait loci (QTLs) that control the flowering and maturity of soybeans. The loci, designated as *E* loci, influence the time to flowering and maturity in soybeans. Many studies have identified the maturity loci *E1* to *E11*, *J*, *Tof5*, *Tof11*, and *Tof12* in soybeans [21,22,23,24,25,26,27,28,29,30,31,32,33]. Among these loci, the dominant alleles of *E1*, *E2*, *E3*, *E4*, *E7*, *E8*, and *E10* were associated with late flowering, whereas those of *E6*, *E9*, *E11*, and *J* showed early flowering in soybeans. Additionally, maturity loci, such as *E1* (*Glyma.06G207800*), *E2* (*Glyma.10G221500*), *E3* (*Glyma.19G224200*), *E4* (*Glyma.20G090000*), *E9* (*Glyma.16G150700*), *E10* (*Glyma.08G363100*), *J* (*Glyma.04G050200*), *Tof5* (*Glyma.05G157300*), *Tof11* (*Glyma.U034500*), and *Tof12* (*Glyma.12G073900*), have been molecularly characterized in the soybean genome. Studies have been conducted to examine the allelic variation in maturity genes for flowering and maturity in different geographic regions in China and Japan through the genotyping of *E1*, *E2*, *E3*, and *E4* [28,30,31,32]. These studies revealed that later-maturing lines had *E1*, *E2*, *E3*, and *E4* genotypes, whereas early-flowering lines showed two or three recessive alleles of these maturity genes. Additionally, allele combinations of three cloned *E* genes (*E1*, *E2*, and *E3*) are explained by different MGs in the US [34]. The development of cultivars in specific regions in China, Japan, and the US is important for using maturity genes through marker-assisted selection.

Korea is the center of origin for wild soybean with a long history of soybean cultivation [35]. There are ~17,000 *G. max* and ~3700 *G. soja* accessions in Korea [36]. In this study, we used 1467 wild soybean accessions as the total population. This study aimed to construct a core subset of 1467 wild soybean accessions and analyze their genetic diversity using 180 K Axiom^®^ SoyaSNP to understand their genetic variations [37]. Additionally, genome-wide association studies (GWASs) were conducted to detect the genetic loci underlying time to flowering for a core subset collection, and they revealed the allelic variation in *E* genes (*E1*, *E2*, and *E3*) using the available resequencing data of wild soybeans.

## 2. Results

### 2.1. Construction of the Core Collection

**Figure 1:**
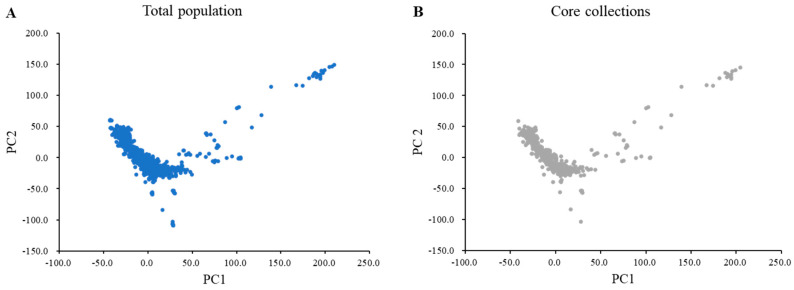
Principal component analysis of the total population and core collection with 180 K Axiom^®^ SoyaSNP. (**A**) Blue indicates the total wild soybean accessions (n = 1467). (**B**) Gray represents 408 selected core collections.

In total, 170,233 SNPs from 1467 wild soybean collections were used to construct the core collection. The core collections included 408 wild soybeans, accounting for 27.8% of the total population. The core subset of the wild soybean collection represented 99.0% of the genetic diversity of the 1467 wild soybean collections. A principal component analysis (PCA) showed that the distribution of the core subset of collections covered the total population based on the first PC (PC1) and the second PC (PC2) (Figure 1). The core subset of collections evenly covered the total population of PC1 and PC2. The variances of PC1 and PC2 were 19.37% and 10.09%, respectively. A wild core subset of collections consisting of 408 wild soybeans was constructed and used for subsequent analyses.

### 2.2. Genetic Diversity and Population Structure

**Figure 2:**
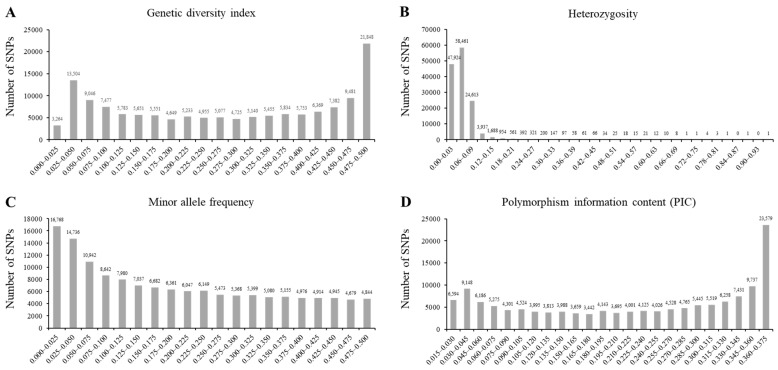
The genetic diversity analyses of core subset of wild soybean collections. Genetic diversity index with 180 K SNP genotype (**A**), heterozygosity with 180 K SNP genotype (**B**), minor allele frequency with 180 K SNP genotype (**C**), and the polymorphism information content (PIC) (**D**) of 142,177 SNPs across 408 wild soybean collections with green cotyledon and 3 cultivars.

The 180 K single-nucleotide polymorphism (SNP) markers were used to evaluate the genetic diversity indices of the wild soybean collection. The genetic diversity index ranged from 0.022 to 0.500. The mean of the genetic diversity index was 0.272 (Figure 2A). Approximately 97.8% of the analyzed SNP markers showed a heterozygosity value of <0.09 (Figure 2B). The minor allele frequencies ranged from 0.011 to 0.555, and the mean value was 0.197 (Figure 2C). The polymorphism information content (PIC) indicated the allelic diversity of the evaluated SNP markers (Figure 2D). The maximum and minimum PIC values were 0.375 and 0.022, respectively, with an average of 0.222. According to the analysis of the genetic diversity indices in this study, 119 SNPs across 20 chromosomes showed the highest diversity among the 180 K SNP genotypes.

**Figure 3:**
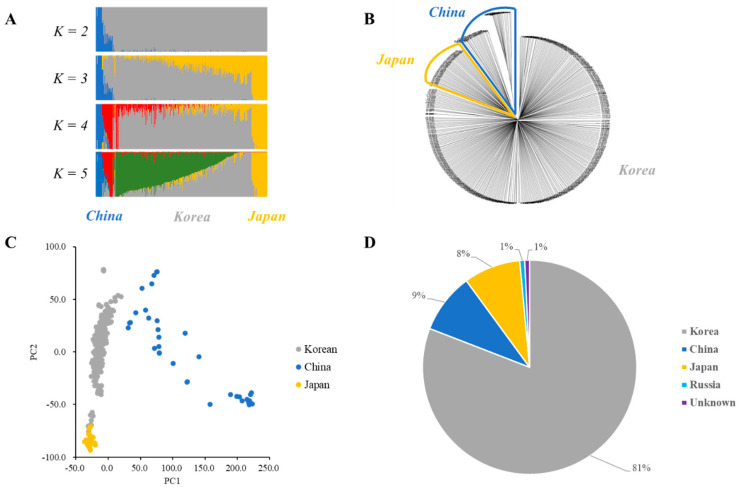
Cluster analyses and a phylogenetic tree of 408 wild soybean accessions of the core subset. (**A**) The admixture plot. Clustering of *K* values from 2 to 5 for wild soybean core collections. Each wild soybean accession is shown on a vertical bar, representing the proportion of the genome of the wild soybean accession from the clusters. (**B**) Unweighted pair group method with arithmetic mean (UPGMA) phylogenetic tree of 408 wild soybean accessions. Blue represents the Chinese wild soybean, and yellow represents the Japanese wild soybean collections. (**C**) Principal components of SNP variation. The Korean, Chinese, and Japanese clusters are indicated in gray, blue, and yellow, respectively. (**D**) Distribution of three clusters in the core subset.

Population clustering was analyzed using fastSTRUCTURE with the wild soybean collection regions. Admixture plots of *K* values from 2 to 5 were constructed (Figure 3A). The marginal likelihood increased with an increase in the *K* value. Additionally, PCA and the unweighted pair group method with an arithmetic mean (UPGMA) phylogenetic tree analysis were shown to determine the optimal number of clusters, supporting the fastSTRUCTURE results (Figure 3B,C). Therefore, 408 wild soybean collections in the core subset could be divided into 3 clusters (*K* = 3) (Figure 3). Cluster 1 comprised 333 wild soybean accessions, consisting of 327 Korean, 3 Russian, and 3 unknown wild soybean accessions (Figure 3D). In contrast, Cluster 2 comprised 37 Chinese wild soybean accessions, and Cluster 3 consisted of 35 Japanese and 3 Korean wild soybean collections. Clusters 1, 2, and 3 of the core collections were mainly collected from Korea, China, and Japan, respectively (Figure 3D). Manhattan plots of the F_st_ estimation between each cluster are shown in Figure S1 and Table S1. The F_st_ estimations between each cluster were 0.114, 0.126, and 0.049 between Cluster 1–Cluster 2, Cluster 1–Cluster 3, and Cluster 2–Cluster 3, respectively (Table S1).

**Figure 4:**
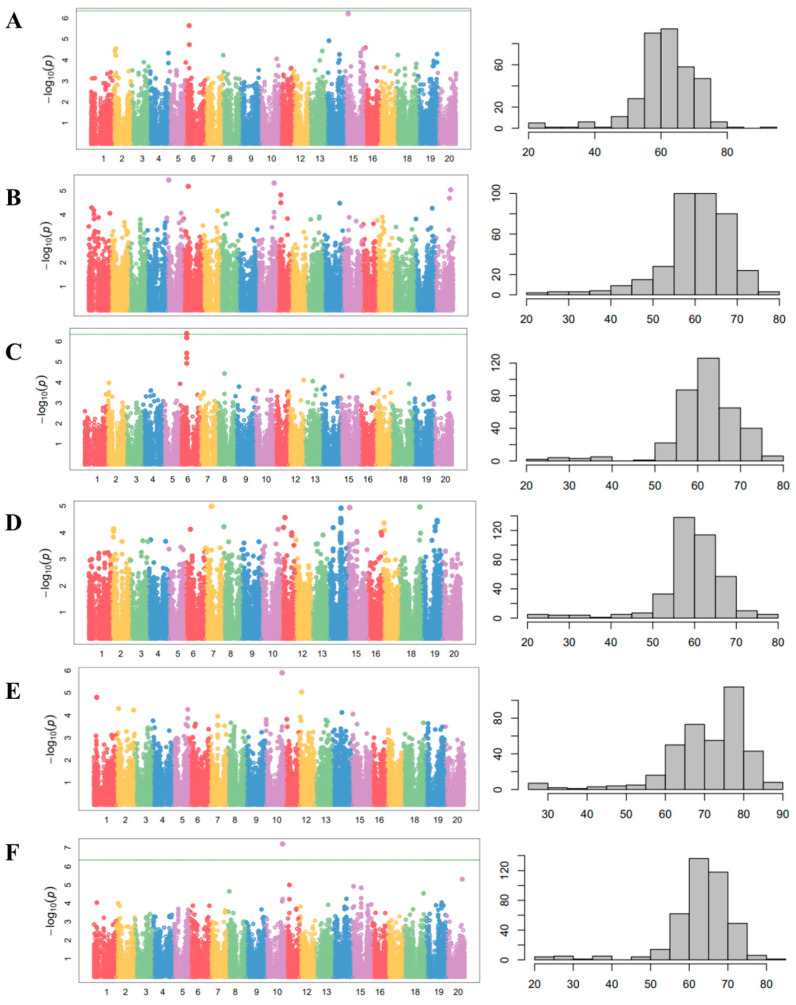
Manhattan plots of CMLM analysis and phenotypic distribution for days to flowering with 408 wild soybean accessions in 5 different environments. (**A**) Suwon, 2012. (**B**) Suwon, 2013. (**C**) Gwangju, 2016. (**D**) Jeonju, 2016. (**E**) Ochang, 2016. (**F**) The mean of five environments. The horizontal green line indicates the genome-wide significance threshold based on Bonferroni correction (*p* < 0.05).

The phenotypic distribution of the 408 wild soybean accessions for the days to flowering was shown in 5 different environments based on 3 clusters (Figure 4). The Chinese wild soybean accessions had significantly earlier flowering than the Korean and Japanese wild soybeans in five different environments. The wild soybean collections from Korea and Japan showed similar days to flowering in three environments (Suwon in 2012, Suwon in 2013, and Ochang in 2016). However, the Japanese wild soybean collections had significantly later flowering than the Korean wild soybean accessions at Jeonju and Gwangju in 2016.

### 2.3. Phenotypic Distribution and GWAS for Days to Flowering with a Core Population

**Figure 5:**
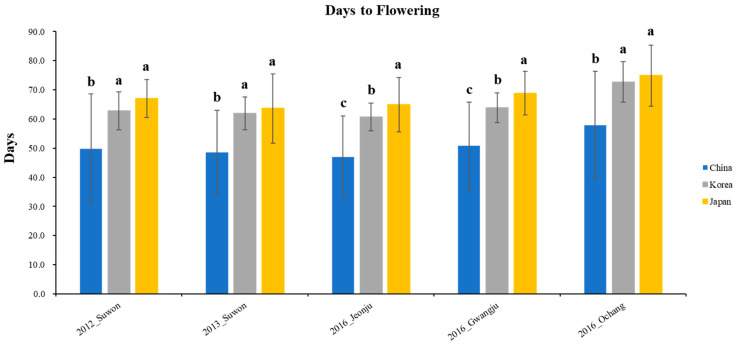
Phenotypic distribution for days to flowering with 408 core collections in 5 environments. The Korean, Chinese, and Japanese clusters are indicated in gray, blue, and yellow, respectively. Bars indicate standard deviation. LSD is the least square difference between clusters in five different environments, and different characters on bars indicate 5% significance level.

The phenotypic distribution of the 408 wild soybean accessions for the days to flowering was measured in 5 different environments (Figure 5) to understand the association between phenotype and QTL. The days to flowering were measured as the number of days from planting to the beginning of bloom. The days to flowering of the wild soybean accessions at Suwon in 2012 ranged from 21 to 92 days, with an average of 62.1 days. The days to flowering at Suwon in 2013 ranged from 24 to 79 days, with an average of 60.8 days. Additionally, the days to flowering at Jeonju, Gwangju, and Ochang in 2016 were 59.5 ± 8.3, 62.7 ± 8.4, and 71.2 ± 10.4 days, respectively. The ranges of days to flowering at Jeonju, Gwangju, and Ochang in 2016 were 24–80, 22–80, and 28–88 days, respectively.

A GWAS was performed using a compressed mixed linear model (CMLM) and 142,177 SNPs (Figure 4). The summarized results of the CMLM analyses with the days to flowering in five environments and the SNPs that reached a value of >5.0 on a −log_10_(*p*) scale are represented in Table 1. Among the SNPs in Table 1, the SNP on chromosome 6 (AX-90416460), the SNP on chromosome 10 (AX-90408467) at Gwangju in 2016 (Figure 4D), and the means of five environments (Figure 4F) were significantly associated with the days to flowering. A haplotype analysis revealed that the SNPs on chromosome 10 across three environments, namely, Suwon in 2013, Gwangju in 2016, and the means of five environments, were in the *E2* locus.

### 2.4. Allelic Variation in E Genes in Publicly Available Genome Sequencing Data

Our previous study revealed the genome sequences of wild soybeans, which are available in the National Center for Biotechnology Information [38]. Overall, the resequencing data of 334 wild soybean accessions from a core population in this study were used (available online). The allelic variations in *E1* (*Glyma.06G207800*), *E2* (*Glyma.10G221500*), and *E3* (*Glyma.19G224200*) were analyzed using the genome sequencing data of wild soybeans to reveal the allelic variation in *E* genes (*E1*, *E2*, and *E3*) for predicting maturity in the wild soybean core collections (Figure 5).

**Figure 6:**
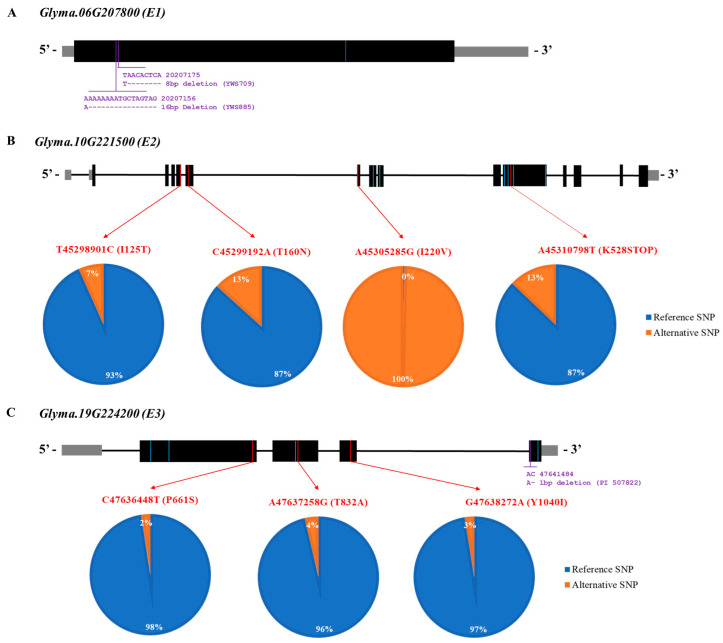
Gene structures of *E1*, *E2*, and *E3* are shown with their variants of sequenced wild soybean accessions. (**A**) Gene model of *Glyma.06G207800* (*E1*), where black boxes indicate exon regions, gray boxes indicate the untranslated region, and black lines indicate the intron region. Blue color indicates a silent mutation of *E1*. (**B**) Gene model of *Glyma.10G221500* (*E2*), where black boxes indicate exon regions, gray boxes indicate the untranslated region, and black lines indicate the intron region. Missense and nonsense mutations in the exon regions are marked with red lines. Blue indicates a silent mutation of *E2*. The reference SNP indicates *the Glycine max* Wm82.a2 reference genome. The pie charts indicate the proportion of reference and alternative SNPs for missense and nonsense mutations. Blue color indicates the reference SNP which is W82.a2.v1, while orange color indicates the alterative SNP which is different from SNP of Williams 82 (**C**) Gene model of *Glyma.19G224200* (*E3*), where black boxes indicate exon regions, gray boxes indicate the untranslated region, and black lines indicate the intron region. Missense mutations in the exon regions are marked with red lines. Blue indicates a silent mutation of *E3*. The proportions of reference and alternative SNPs are shown in pie charts. Blue color indicates the reference SNP which is W82.a2.v1, while orange color indicates the alterative SNP which is different from SNP of Williams 82.

The SNP variants at C20,207,605T causing a silent mutation are shown as a blue line on the *E1* gene in Figure 6A. Ten collections had thymine at position 20,207,605 (Table S2). For the INDEL, two deletions were identified in wild soybean accessions: YWS709 (8 bp deletion) and YWS885 (16 bp deletion). There were 10 SNP variants for the *E2* gene consisting of 3 missense, 1 nonsense, and 6 silent mutations in 6 different exons of the *E2* gene: T–C at physical position 45,298,901, C–A at 45,299,192, A–G at 45,305,285, G–T at 45,305,867, T–C at 45,306,065, A–G at 45,310,665, C–T at 45,310,713, A–T at 45,310,798, A–T at 45,310,896, and C–T at 45,312,060 (Figure 6B). The red lines of the SNP variants in the figure represent missense and nonsense mutations, whereas the blue lines of the SNP variants indicate silent mutations. Twenty-two wild soybean accessions (7.0%) had an SNP variant at position T45,298,901C, resulting in an isoleucine-to-threonine variant at amino acid position 125 of the *E2* gene. Approximately 13.0% of the sequenced wild soybean accessions had an SNP variant at position C45,299,192A (T160N). Interestingly, all sequenced wild soybean accessions showed 100% SNP at position A45,305,285G (I220V). Nonsense mutations at A45,310,798T were detected in 13.0% of the sequenced wild soybean accessions in the *E2* gene. In the *E3* gene, there were seven SNPs with three missense (red line) and four silent (blue line) mutations (Figure 6C). Three missense mutations on the *E3* gene were revealed at positions C47,636,448T (P661S), A47,637,258G (T832A), and G47,638,272A (Y1040I). A wild soybean, PI 507822, contained one 1 bp deletion at position 47,641,485, based on the INDEL information.

## 3. Discussion

The current study used 1467 wild soybean collections from the National Agrobiodiversity Center of South Korea to construct 408 Korean wild core collections based on 180 K Axiom^®^ SoyaSNP genotypic information. Although wild soybean germplasms increase genetic diversity and have favorable genes [3,4], it is challenging to grow wild soybean because of its undesirable traits, such as lodging, viny growth habits, hard seed coats, and pod shattering. Constructing a core subset is one of the strategies to overcome the handling of a large population of wild soybean accessions. In this study, the 180 K SNP markers were analyzed to directly reflect the genetic diversity of the total population, in contrast to phenotypic observations that are primarily influenced by environmental factors. Our previous study revealed 430 Korean core collections of *G. max* from 2872 collections based on the 180K SNP genotype [37,38,39,40]. Additionally, we recently provided resequencing data on the core collection of *G. max* and wild soybeans [38]. The Korean core collection of *G. max* and wild soybeans as diverse genetic resources can be used for genetic and genomic analyses in soybean breeding programs.

Studies have shown that the proportion of the core collection from the total population generally ranges from 5% to 20% and 99.0% of the genetic diversity of the total population [39,41]. This study revealed that 408 wild soybeans as the core collection can be useful genetic materials instead of growing 1467 soybean collections in wild soybean research. The core collection was evenly distributed in the total population of PC1 and PC2 (Figure 1). Additionally, phylogenetics and PCA revealed that three clusters of core collections mainly represented the collection regions Korea, China, and Japan. The extent of genetic differentiation between each cluster is shown using the F_st_ statistic (Table S1). The Chinese wild soybean collections (Cluster 1) in this study showed a higher level of population differentiation from the Korean (Cluster 2) and Japanese wild soybean collections (Cluster 3). The F_st_ value between Cluster 2 and Cluster 3 was the smallest, suggesting that they were similar to each other. Therefore, the 408 Korean wild soybean accessions of the core subset in this study are helpful genetic germplasms for genetic studies and soybean breeding programs and for exploring candidate genes related to traits through GWASs.

GWAS analyses were conducted with 180 K Axiom^®^ SoyaSNP to identify the loci in the wild soybean for the days to flowering of the 408 core collections. Although we did not detect significantly overlapped SNPs in five different environments, two SNPs on chromosome 6 (AX-90416460), chromosome 10 (AX-90408467) at Gwangju in 2016 (Figure 5D), and the mean of five environments (Figure 5F) were significantly associated with the days to flowering. The SNPs on chromosome 6 were near the *E1* locus at Gwanju in 2016, and most of the wild soybeans contained a functional *E1* gene, except for two wild soybean accessions with deletions (Figure 6A). The SNPs on chromosome 10 across three environments (Suwon in 2013, Gwangju in 2016, and the mean of five environments) were in the *E2* locus. All 388 wild soybean collections contained a missense mutation at A45,305,285G (I220V) on the *E2* gene, as determined via an analysis of the resequencing data of 388 wild soybean collections, although isoleucine and valine are nonpolar amino acids, meaning that the function of these 2 amino acids is similar. Thus, further research is required to identify the expression level of the *E2* gene in wild soybean accessions.

Maturity *E* genes are highly associated with MGs and the days to maturity in soybeans. Zhai et al. [42] identified 180 soybean cultivars with *E1*, *E2*, *E3*, and *E4* genotypes. The earliest-maturing lines had *e1*, *e2*, *e3*, and *e4* genotypes, and the later-maturing lines had *E1*, *E2*, *E3*, and *E4* genotypes in three northern and southern regions in China. Tsubokura et al. [43] used molecular markers to determine the genotypes of maturity genes and revealed that early-flowering lines had two or three recessive alleles and that late-flowering lines had *E1*, *E2*, *E3*, and *E4* genotypes. Lagewisch et al. [34] proposed the *E* gene maturity model with an allelic variation in the *E1*, *E2*, and *E3* genes to adapt to different MGs in the US and Canada. Thus, we analyzed the resequencing data of the wild soybean collections to identify the allelic variation in *E1*, *E2*, and *E3* and predict the days to maturity in this study. However, most of the wild soybeans in the core collection had functional *E1* and mutant alleles of the *E2* gene at position A45,305,285G (I220V). The resequencing of the wild soybean collections revealed a small portion of alternative SNPs at each SNP position in the *E3* gene.

We assumed that other maturity genes were likely to cause the days to flowering phenotype in wild soybean accessions. Recent studies have revealed that the stepwise selection of two homologous pseudo-response regulator genes, *Tof11* and *Tof12*, has played an essential role in soybean growth at high latitudes during domestication [33,44,45]. Additionally, *Tof5* encoded a homolog of *Arabidopsis thaliana* FRUIT-FULL that promotes flowering in soybeans [46]. They reported that *Tof5* for flowering was under parallel selection during soybean domestication. We suggest that these wild soybean collections can be used as genetic materials to identify new maturity or flowering genes for further studies.

In conclusion, 408 wild soybean collections from 1467 accessions were constructed as core wild soybean collections from 180 K SNP genotypic data. These wild soybean collections can be used as genetic materials to identify new maturity or flowering genes and favorable traits for further studies. This core collection can provide helpful genetic resources for developing new cultivars, thereby facilitating the introgression of genes of interest from wild soybeans as part of a breeding program.

## 4. Materials and Methods

### 4.1. Wild Soybean Collections

In our previous study, 1467 wild soybean collections were obtained from the National Agrobiodiversity Center and Yeongnam University, Republic of Korea, to construct wild soybean core collections [40]. Among these, 1343 wild soybean accessions were collected from South Korea, 58 from China, 56 from Japan, and 6 from Russia, and 3 were unknown wild soybean accessions.

### 4.2. DNA Isolation and Genotyping with Axiom^®^ 180k SoyaSNP Array

To isolate the genomic DNA, we collected young trifoliate leaves from each wild soybean collection. Next, the leaf samples were ground into a fine power under liquid nitrogen with a mortar and pestle, and genomic DNA was extracted using the cetyltrimethylammonium bromide (CTAB) method with a minor modification [47]. The quality of the genomic DNA from each wild soybean accession was evaluated using 1.5% agarose gel electrophoresis. Next, 30 μL of 100 ng/µL genomic DNA from the 1467 wild soybean accessions was genotyped using the Axiom^®^ 180k SoyaSNP array [37]. The SNPs were scored based on the Axiom^®^ Genotyping Solution Data Analysis User Guide [48]. In total, 170,233 SNPs were genotyped from 180,961 SNPs using the Axiom^®^ 180k SoyaSNP array.

### 4.3. Construction of a Core Collection

A total of 170,233 SNPs of the 1467 wild soybean accessions were analyzed using GenoCore [49] to construct a core collection. GenoCore was used based on two parameters, coverage, and delta. The coverage provided a percentage value for a core subset representing the total population, and delta represented an increasing coverage ratio. To construct the core subset of the collection with GenoCore, the parameters were set as 99.0% of the coverage and 0.001% of the delta value.

### 4.4. Core Subset for Days to Flowering

Phenotypic observations of the days to flowering in five environments were obtained via field evaluations. A core collection of wild soybeans was tested in an experimental field at the National Institute of Crop Science (Suwon; 37°15′ N, 126°58′ E) in 2012 and 2013. The core collections were planted in 2016 at three locations, namely, an experimental field at the National Institute of Crop Science (Jeonju; 35°50′ N, 127°02′ E), affiliated experiment and practice fields of Chonnam National University (Gwangju; 36°17′ N, 126°39′ E), and a field at the Korea Research Institute of Bioscience and Biotechnology (Ochang; 36°43′ N, 127°26′ E). Seedlings were germinated in trays and transplanted onto experimental fields using hand-on hills in an area of 1.5 × 0.8 m. Each plot grown was harvested in bulk at the full maturity of the plant (R8) [50].

### 4.5. Population Structure of a Core Subset including 408 Wild Soybean Accessions

With PowerMarker 3.25 [51], we obtained diversity indices, such as minor allele frequencies, the genetic diversity index, PIC, and heterozygosity, using 408 wild soybean collections with 142,177 SNP markers. PCA was conducted with the 1467 wild soybean accessions and a core subset using the R package SNPRelate [52]. The PCA plot showed PC1 against PC2. For the phylogenetic tree in this study, an UPGMA tree was constructed with all wild soybean accessions using the calculation of a modified Euclidean distance between each pair of wild soybean collections, as determined via TASSEL [53]. The admixture plots from *K* = 1 to *K* = 10 were analyzed using fastSTRUCTURE [54]. Genetic differentiation (F_st_) between each cluster was calculated using VCFtools [55].

### 4.6. GWAS for Days to Flowering

To conduct a GWAS, TASSEL and GAPIT were used in this study. A total of 142,177 SNPs were finalized for further analyses after the filtration of SNPs with 20% missing data and a minor allele frequency (0.01) and to construct PCA and the kinship coefficient matrix [56,57]. We used a compressed mixed linear model (CMLM) to generate Manhattan plots [57,58]. The significance threshold of −log_10_(*p*) was determined using Bonferroni’s correction and false discovery rates.

### 4.7. Resequencing Data of 334 Wild Soybean Core Collections

Large datasets, including SNP and INDEL variants of 855 soybean accessions, were available from the figshare repository (https://figshare.com/projects/Soybean_haplotype_map_project/76110 (accessed on 1 December 2022)) [38]. For further analyses, the SNPs and INDEL of 334 wild soybean collections were extracted from 855 resequenced accessions for allelic variation in *E1*, *E2*, and *E3* genes. In total, 334 wild soybean collections belonged to the core subset in this study.

### 4.8. Statistical Analysis

Statistical analyses were conducted using SAS v9.4 (SAS Institute, Cary, NC, USA, 2013). Mean differences among the genotypic groups were analyzed using Fisher’s least significant difference test at a *p* value of 0.05 using PROC GLM.

## Figures and Tables

**Table 1:** SNP loci associated with days to flowering in five different environments.

Environments	SNP	Chromosome	Position (Wm82.a2)	−log_10_(*p*)	Minor Allele Frequency	*R*^2^ of Model without SNP	*R*^2^ of Model with SNP	Allelic Effect
Suwon 2012	AX-90393598	6	9,723,612	5.64	0.06	0.48	0.51	−6.78
	AX-90341967	15	7,724,810	6.21	0.05	0.48	0.51	4.80
Suwon 2013	AX-90364087	5	4,972,021	5.45	0.46	0.49	0.52	1.63
	AX-90450038	6	13,556,490	5.19	0.11	0.49	0.52	2.62
	AX-90408467	10	42,924,587	5.33	0.07	0.49	0.52	−5.33
	AX-90501757	20	40,976,128	5.04	0.06	0.49	0.52	5.13
	AX-90460646	20	40,976,622	5.04	0.06	0.49	0.52	−5.13
Gwangju 2016	AX-90416460	6	17,937,235	6.39	0.06	0.65	0.67	−3.61
	AX-90438603	6	17,945,147	6.18	0.06	0.65	0.67	−3.42
	AX-90440430	6	17,965,684	5.43	0.08	0.65	0.67	−2.71
	AX-90518769	6	17,966,038	5.20	0.08	0.65	0.67	−2.69
Ochang 2016	AX-90408467	10	42,924,587	5.90	0.06	0.58	0.61	−6.40
	AX-90382309	12	6,676,921	5.03	0.11	0.58	0.60	2.77
Mean	AX-90408467	10	42,924,587	7.25	0.06	0.69	0.72	−4.85
	AX-90460646	20	40,976,622	5.18	0.07	0.69	0.71	−3.71
	AX-90501757	20	40,976,128	5.18	0.07	0.69	0.71	3.71

## Data Availability

The datasets generated during this study are available from the corresponding author upon reasonable request.

## Supplementary Materials

The following supporting information can be downloaded at: https://www.mdpi.com/article/10.3390/plants12061305/s1, Figure S1: Manhattan plot of F_st_. Manhattan plot of genome-wide F_st_ between clusters against the position of SNPs on each of the 20 chromosome. (A) Manhattan plot of F_st_ between cluster 1 and cluster 2. (B) Manhattan plot of F_st_ between cluster 1 and cluster 3. (C) Manhattan plot of F_st_ between cluster 2 and cluster 3; Figure S2: Number of SNPs within 1 Mb window for all the 20 chromosomes; Table S1: F_st_ values between clusters; Table S2: Allelic variation of *E* genes based on re-sequencing of wild soybean accessions.
