# Supplementary material for: Genetic Diversity of Korean Wild Soybean Core Collections and Genome-Wide Association Study for Days to Flowering
Source: Plants (Basel). 2023 Mar 14;12(6):1305. doi: 10.3390/plants12061305 (PMC10058364; doi:10.3390/plants12061305)
Supplement: Supplementary file 1 [file plants-12-01305-s001.zip › Supplementary figures.pdf]

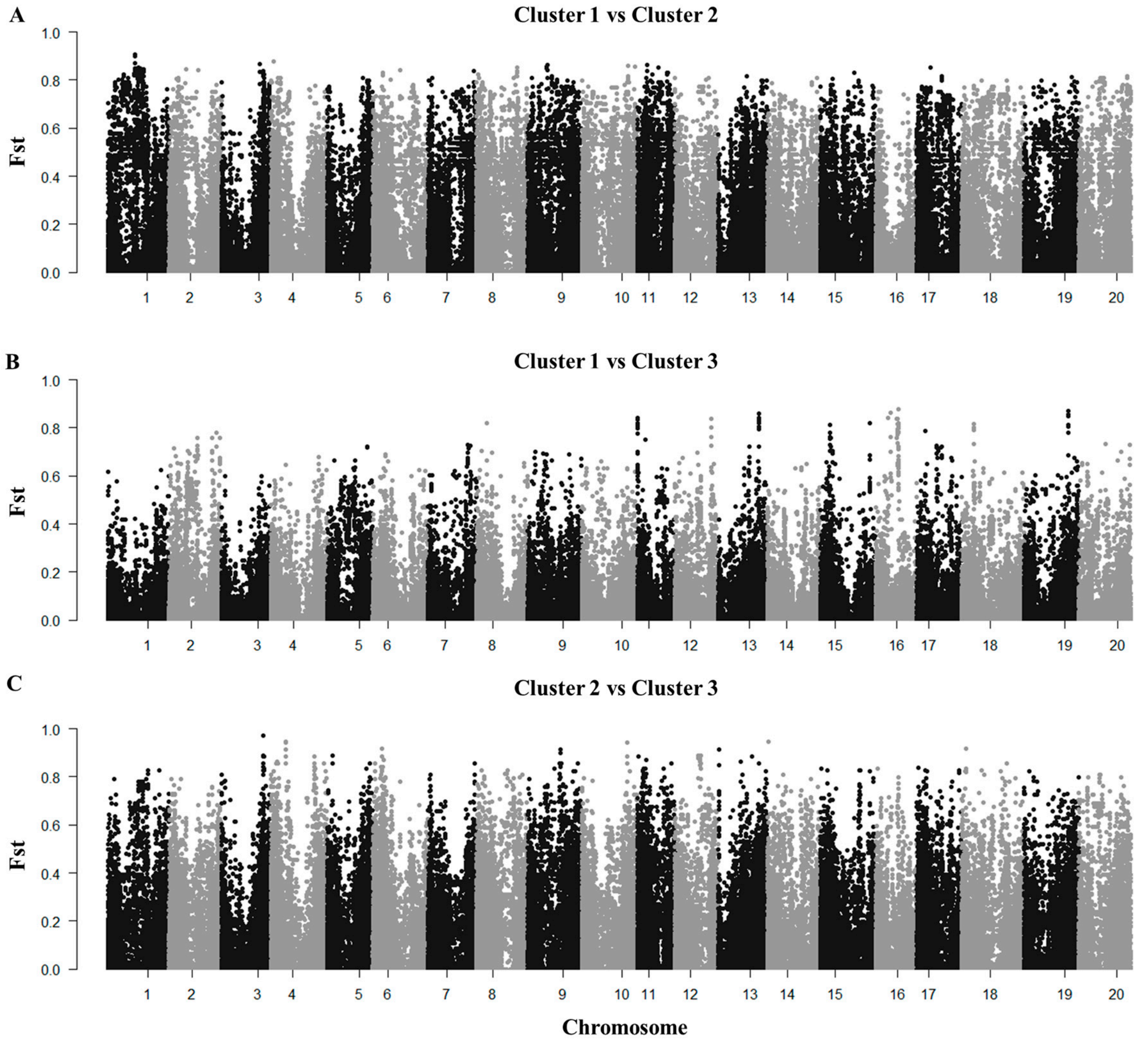

Figure S1. Manhattan plot of Fst. Manhattan plot of genome-wide Fst between clusters against the position of SNPs on each of the 20 chromosome. (A) Manhattan plot of Fst between cluster 1 and cluster 2. (B) Manhattan plot of Fst between cluster 1 and cluster 3. (C) Manhattan plot of Fst between cluster 2 and cluster 3.

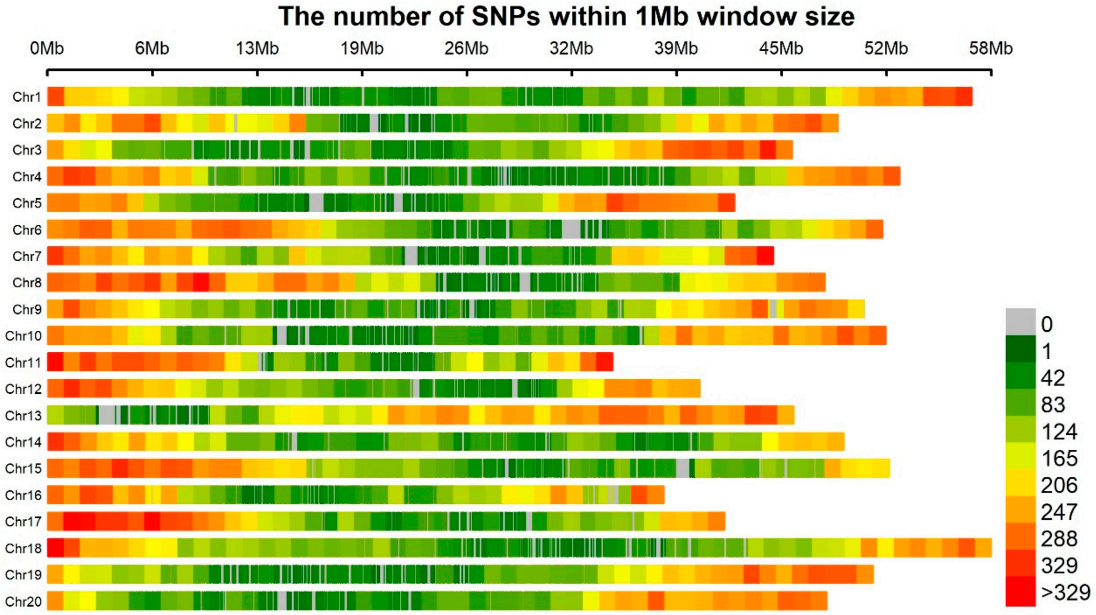

Figure S2. Number of SNPs within 1 Mb window for all the 20 chromosomes.
